# Thermal Stability and Vibrational Properties of the 6,6,12-Graphyne-Based Isolated Molecules and Two-Dimensional Crystal

**DOI:** 10.3390/ma16051964

**Published:** 2023-02-27

**Authors:** Ekaterina S. Dolina, Pavel A. Kulyamin, Anastasiya A. Grekova, Alexey I. Kochaev, Mikhail M. Maslov, Konstantin P. Katin

**Affiliations:** 1 Institute of Nanotechnologies in Electronics, Spintronics and Photonics, National Research Nuclear University “MEPhI”, Kashirskoe Sh. 31, Moscow 115409, Russia; 2 Laboratory of Computational Design of Nanostructures, Nanodevices, and Nanotechnologies, Research Institute for the Development of Scientific and Educational Potential of Youth, Aviatorov Str. 14/55, Moscow 119620, Russia; 3 Laboratory of Acoustic Microscopy, Science Institute of Biochemical Physics Named after N.M. Emanuel of the Russian Academy of Sciences, Kosygina Str. 4, Moscow 119334, Russia; 4 Research and Education Center “Silicon and Carbon Nanotechnologies”, Ulyanovsk State University, Leo Tolstoy Str. 42, Ulyanovsk 432017, Russia

**Keywords:** 6,6,12-graphyne, radiaannulenes, molecular dynamics, thermal stability, Raman and IR spectra

## Abstract

We report the geometry, kinetic energy, and some optical properties of the 6,6,12-graphyne-based systems. We obtained the values of their binding energies and structural characteristics such as bond lengths and valence angles. Moreover, using nonorthogonal tight-binding molecular dynamics, we carried out a comparative analysis of the thermal stability of 6,6,12-graphyne-based isolated fragments (oligomer) and two-dimensional crystals constructed on its basis in a wide temperature range from 2500 to 4000 K. We found the temperature dependence of the lifetime for the finite graphyne-based oligomer as well as for the 6,6,12-graphyne crystal using a numerical experiment. From these temperature dependencies, we obtained the activation energies and frequency factors in the Arrhenius equation that determine the thermal stability of the considered systems. The calculated activation energies are fairly high: 1.64 eV for the 6,6,12-graphyne-based oligomer and 2.79 eV for the crystal. It was confirmed that the thermal stability of the 6,6,12-graphyne crystal concedes only to traditional graphene. At the same time, it is more stable than graphene derivatives such as graphane and graphone. In addition, we present data on the Raman and IR spectra of the 6,6,12-graphyne, which will help distinguish it from the other carbon low-dimensional allotropes in the experiment.

## 1. Introduction

Graphene is a hexagonal monolayer of carbon atoms with extraordinary flexibility, stability, and electrical and thermal conductivity, making it highly interesting as a promising material for carbon-based electronics, photonics, and optoelectronics [1]. Furthermore, graphene has an extremely high electric conductivity [2], and it is also one of the strongest known two-dimensional materials, which is, at the same time, quite flexible. Since the synthesis of graphene, many other two-dimensional materials have been discovered or proposed, including graphone [3], graphane [4], graphene oxide [5], phagraphene [6], graphyne [7], etc., which are of interest due to their unique atomic structure, as well as mechanical, optical, and electronic properties [8,9]. For example, graphane and graphone are hydrogenated derivatives of graphene. If graphane is a fully (on both sides) hydrogenated sheet of graphene, then graphone is semi-hydrogenated graphene, covered with hydrogen atoms only on one side. Graphene oxide is a layered carbon structure with oxygen-containing functional groups attached to both sides of the layer as well as the edges of the plane. Finally, phagraphene is a proposed graphene allotrope composed of five-, six-, and seven-membered carbon rings. It should be noted that a wide family of graphene-like two-dimensional materials are considered promising for various applications, including optoelectronic devices, biomedicine, biosensors, energy conversion, and storage [10,11,12,13].

**Figure 1:**
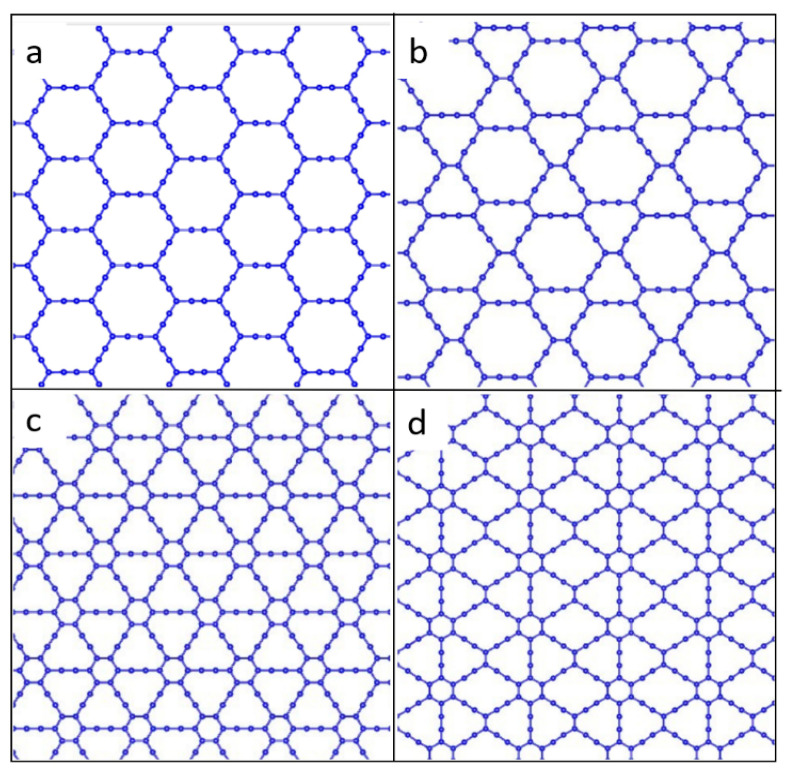
Atomic structure of four types of graphyne: (**a**) α-graphyne, (**b**) β-graphyne, (**c**) γ-graphyne, and (**d**) 6,6,12-graphyne.

Graphyne is a two-dimensional carbon allotrope consisting of sp and sp^2^-hybridized carbons. Graphyne differs significantly in its structure and properties from other allotropic modifications of carbon [14]. Four main types of graphyne were identified. They are α-graphyne, β-graphyne, γ-graphyne, and 6,6,12-graphyne, each with a different percentage of acetylene bonds. These graphyne types are shown in Figure 1. For α-graphyne, all sp^2^-bonds in graphene were replaced by acetylene bonds. The rate of acetylene bonds is 66.67% for β-graphyne, 41.67% for 6,6,12-graphyne, and 33.33% for γ-graphyne [15].

**Figure 2:**
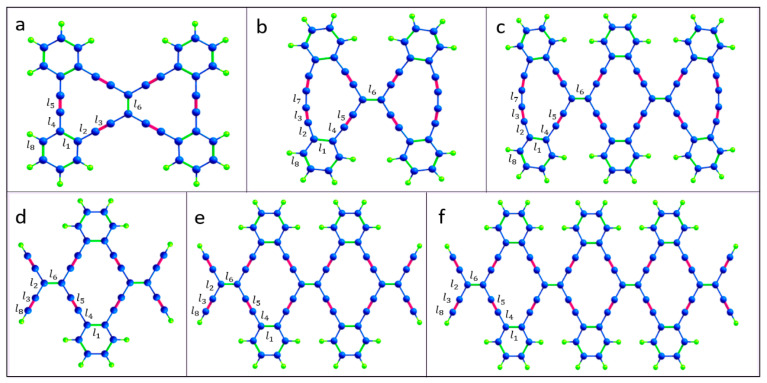
Atomic structure of the isolated 6,6,12-graphyne fragments (**a**) O1, (**b**) O2, (**c**) O3, (**d**) O4, (**e**) O5, and (**f**) O6. The edges are passivated with hydrogen atoms. Carbon and hydrogen atoms are represented by blue and green colors, respectively.

In some respects, graphyne has properties superior to those of graphene [16,17]. Of particular interest is 6,6,12-graphyne, as it established that hexagonal symmetry is not a prerequisite for the existence of graphene-like Dirac fermions. The uniqueness lies in the fact that the 6,6,12-graphyne has a directed anisotropy in the mobility of carriers [15]. In addition, the energy levels of the conduction electrons of the graphyne form the distorted Dirac cones. The properties of graphyne are promising for several applications, such as transistors, sensors, anisotropic conductors, and hydrogen storage [18]. Although the two-dimensional crystal of graphyne has not yet been synthesized, isolated fragments of 6,6,12-graphyne have recently been obtained in the form of hydrocarbon oligomers called radiaannulenes [19]. Some of them are shown in Figure 2.

**Figure 3:**
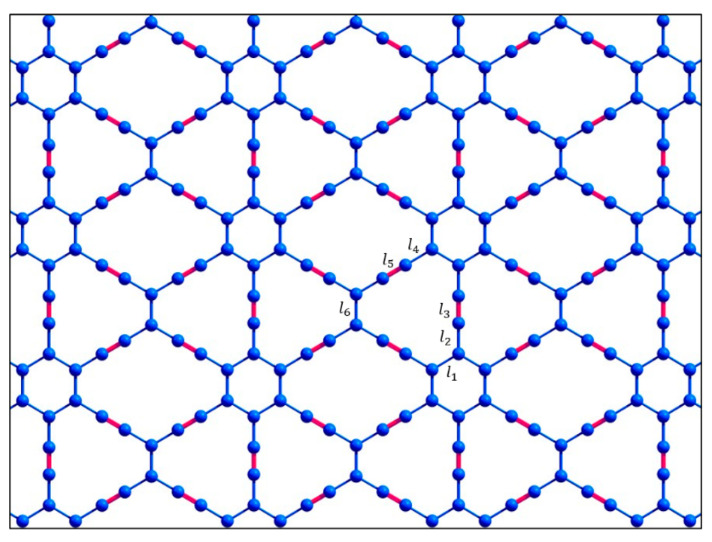
Atomic structure of the two-dimensional 6,6,12-graphyne sheet.

In a recent study [17], the structural, vibrational, electronic, and optical properties of such 6,6,12-graphyne oligomers and their possible applications were studied using density functional theory. Thus, their structural characteristics have been predicted, some quantum chemical descriptors have been described, and Raman and UV-vis spectra have been obtained [17]. However, two-dimensional 6,6,12-graphyne crystals (Figure 3) have not been studied enough.

Thus, the main purpose of this study is to evaluate the thermal stability of 6,6,12-graphyne and compare it with the individual oligomers and with previously obtained data for graphene and other carbon allotropes [20,21]. We were able to demonstrate that the 6,6,12-graphyne crystal and its finite oligomers predicted earlier possess high thermal stability comparable to graphene and graphane, which potentially allows them to be used in various technical applications without extreme temperature conditions. For this purpose, we carried out the molecular dynamics calculations of the smallest O4 oligomer (see Figure 2), which can be considered as the elementary block for constructing the two-dimensional 6,6,12-graphyne crystal, and the crystal of 6,6,12-graphyne itself using the periodic boundary conditions, in a wide temperature range. We determined the activation energy and frequency factor in the Arrhenius law and estimated the lifetime of 6,6,12-graphyne crystal at various temperatures. We also obtained the values of the binding energies and structural characteristics of 6,6,12-graphyne such as bond lengths and valence angles. In addition, as part of the study, using density functional theory, the Raman and infrared spectra of both an oligomer and a 6,6,12-graphyne crystal were determined with the aim of further identifying them in the experiment.

## 2. Computational Details

To analyze the thermal stability of the considered systems, we used the molecular dynamics technique with the nonorthogonal tight-binding model (NTBM) [22,23]. This approach requires significantly fewer computer resources than ab initio methods and therefore allows one to study the evolution of a system of ~100 atoms for a long time, 1 ns–1 μs, which is sufficient to collect the necessary statistics. In the nonorthogonal tight-binding model, the total potential energy of the system is the sum of the quantum-mechanical electronic energy and the “classical” ionic repulsive energy. The former is defined as the sum of one-electron energies over the occupied states. The energy spectrum is determined from the stationary Schrödinger equation (2S, 2P_x_, 2P_y_, and 2P_z_ orbitals of carbon atoms and 1S orbitals of hydrogen atoms are considered). The electronic Hamiltonian is calculated from overlap matrix elements using the extended Hückel approximation with Anderson’s distant dependence for the Wolfsberg–Helmholtz parameter. The overlap integrals are calculated using the standard Slater–Koster–Roothaan procedure. Therefore, the analytical expressions for the overlap integrals greatly simplify the calculation of the Hamiltonian, which significantly speeds up the computer simulation of relatively large systems. In addition, second-order differentiation of the expression makes it possible to obtain the Hessian, which is necessary for optimizing the structure and calculating natural oscillation frequencies. In addition, this allows one to get the Hessian, which is necessary for optimizing the structure and calculating vibrational frequencies. The parameter fitting of the nonorthogonal tight-binding model is based on the criterion of the best correspondence between the computed and experimental (not derived from ab initio calculations) values of binding energies, bond lengths, and valence angles of several selected small hydrocarbon molecules (for parametrization details, see [22,23]). To validate the model developed, we computed the binding energies, bond lengths, and valence angles for various H–C–N–O compounds not used in the fitting procedure and compared them with the experimental data. The resulting tight-binding potential is well suited to modelling various H_k_C_l_N_m_O_n_ molecules, from small clusters to large systems, such as graphene, diamond, peptides, polyprismanes, and so forth [22,23,24]. It should be noted that ab initio methods (for example, density functional theory) are very accurate but computer-resource demanding and hence applicable to small systems only. Calculation time within the density functional approach is estimated as ~N^3^-N^4^, where N is the number of atoms in the molecular system.

We performed a numerical simulation at the constant total energy of the system (the sum of its potential and kinetic energies). Such a formulation of the problem corresponds to the situation when the system is not in thermal equilibrium with the environment. In this case, the “dynamic temperature” *T* is a measure of the energy of relative motion of the atoms and is calculated by the formula

(1) $$<E_{kin}>=\frac{1}{2}k_{B}T\left(3N_{at}-6\right)$$

where $<E_{kin}>$ is the time-averaged kinetic energy of the O4 oligomer or 6,6,12-graphyne crystal, *k_B_* is the Boltzmann constant, and *N_at_* is the number of atoms in the oligomer or the crystal unit cell. We considered that the oligomer, in general, does not move or rotate. Therefore, the number of degrees of freedom decreases by six. Note that for the crystal, the number of degrees of freedom naturally decreases by three. Angle brackets in Equation (1) correspond to averaging over 10^3^–10^4^ steps of molecular dynamics. At the initial instant of time, random velocities and displacements were given to each of the atoms in such a manner that the momentum and the angular momentum of the whole system were equal to zero. Then, we calculated the forces that act on the atoms. The classical Newton equations of motion were numerically integrated using the velocity Verlet method. The time step was 3 ps. It should be noted that the velocity Verlet algorithm is conservative concerning the momentum and the angular momentum; for instance, the relative change in the total energy of the systems studied does not exceed 10^−4^ for at least 2 × 10^9^ molecular-dynamic steps. Within the framework of this approach, when the total energy of the system remains constant, which corresponds to the microcanonical ensemble, its “dynamic temperature” *T* decreases upon transition to an energetically less favorable configuration and increases upon transition to an energetically more favorable configuration. To accumulate meaningful statistical data sufficient for determining the temperature dependence of *τ*, calculations were performed in a wide temperature range, *T* = 2500–4000 K. All calculations performed correspond to different sets of initial velocities and displacements.

The thermal stability of the systems studied is characterized by the activation energy—in other words, the minimum energy required to initiate the process of decomposition. The activation energy *E_a_* and frequency factor A for the decomposition process were determined from the temperature dependence of the lifetime of the system using the Arrhenius formula:

(2) $$\tau^{-1}\left(T\right)=Aexp\left(-\frac{E_{a}}{k_{B}T}\right)$$

To initially obtain the equilibrium geometry of the structure, we used the method of structural relaxation. In this case, the corresponding initial configuration relaxed to a state with the local or global energy minimum under the action of intramolecular forces only. First, the forces acting on all atoms were calculated using the Hellmann–Feynman theorem. Next, atoms were shifted in the direction of the forces obtained proportional to the corresponding forces. Then, the relaxation step was repeated. The whole structure was relaxed up to residual atomic forces smaller than 10^−8^  eV/Å.

In addition, for the geometry optimization and study of the optical properties of the isolated oligomer and 6,6,12-graphyne thin film, we employed the implementation of density functional theory (DFT) calculations in GAMESS [25] and the QUANTUM Espresso 6.5 program package [26,27], respectively. For the finite oligomer, the PBE functional [28] and the electron basis set of 6-311G(d,p) [29] were used. For the 6,6,12-graphyne thin film, the plane-wave basis set for valence electron states with a cutoff energy of 140 Ry (1904 eV), corresponding to 640 Ry (8707 eV) for the charge density cutoff, was taken. Local density approximation (LDA) in the Perdew–Wang (PW) functional form for the exchange–correlation energy [30] and Troullier-Martins norm-conserving pseudopotentials [31] for core-electron interactions were used to perform the calculations. The Brillouin zone integrations were performed using the Monkhorst-Pack k-point sampling scheme [32] with the 8 × 8 × 1 mesh grid. Non-physical interactions were eliminated by setting the interlayer vacuum intervals of all structures to 20 Å. Periodical boundary conditions were used.

The atomic equilibrium positions were obtained by the complete minimization of the unit cell using the calculated forces and stress on the atoms. The convergence criterion of self-consistent calculations for ionic relaxations was 10^−10^ eV between two consecutive steps. Furthermore, all atomic positions and the supercell itself were optimized until all components of all forces acting on the atoms became smaller than 10^−4^ hartree/bohr. Such criteria ensured the absolute value of stress was less than 0.01 kbar. To study the Raman and IR spectra, we employed the implementation of the density functional perturbation theory (DFPT) method [33].

## 3. Results and Discussion

### 3.1. Atomic Structure and Energy Characteristics

The resulting oligomer structures and 6,6,12-graphyne, which were obtained after structural relaxation, are shown in Figure 2 and Figure 3. The obtained bond lengths of these structures are listed in Table 1. The length of the C–C bonds in the hexagons is named l_1_ and is equal to 1.46 Å for all oligomer structures. Sp^2^-hybridized bonds of the carbon atoms outside the hexagons have the lengths l_2_, l_4_, and l_6_ as 1.43–1.45 Å. Bonds l_3_ and l_5_ are sp-hybridized bond lengths of the carbon atoms outside the hexagons, which are 1.24–1.25 Å for all studied structures. The bond connecting the two carbon atoms of a triple bond in oligomers O2 and O3 has the length l_7_ as 1.36 Å. The C–H bond length l_8_ is 1.08–1.09 Å.

The values of the valence angles in the structures were also obtained. The internal angles of the butadiene segment of the O2 and O3 oligomers were 170°. The valence angles inside the hexagons were equal for the crystal and all oligomers, 118–122°. For endo- and exocyclic alkylidene angles of 6,6,12-graphyne and oligomers O4, O5, and O6, slight deviations from the value of 120° were observed. Detailed results of calculations of valence angles are given in Supplementary Materials (Figures S1 and S2). The values of the valence angle for the oligomers O4 and O5 can be compared with the values obtained experimentally using X-ray crystallography methods [19]. The data presented in Table 1 show good agreement between the experimental and theoretical results. For example, the difference between the values of bond lengths does not exceed five-hundredths of an angstrom, which may be due to the imperfection of the experimental equipment and the achievable accuracy of numerical calculations. Thus, we can consider that the chosen level of theory for computer simulation of oligomers based on 6,6,12-graphyne is reasonable.

The binding energy values were calculated for the studied structures using the NTBM model and are presented in Table 2.

The configuration corresponding to the maximum binding energy (that is, the minimum potential energy) is the most thermodynamically stable. The obtained results indicate, in general, an increase in the binding energy with an increase in the number of carbon atoms in the structure. Thus, for a two-dimensional 6,6,12-graphyne crystal, the binding energy has a maximum value, which is 6.93 eV/atom. This value corresponds to the binding energy for traditional graphene, which is ~7.3 eV/atom. Thus, we can conclude that the 6,6,12-graphyne crystal possesses stability comparable to that of graphene.

### 3.2. Raman and Infrared Spectra

**Figure 4:**
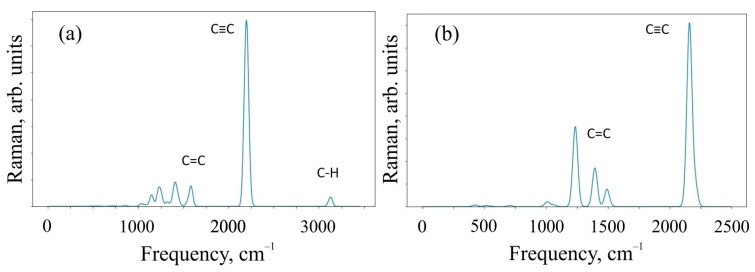
Raman spectra of (**a**) O1 oligomer and (**b**) 6,6,12-graphyne crystal.

To characterize the systems under study, we critically investigated the change in the Raman spectra for the 6,6,12-graphene crystal and its fragment, which was represented by the O1 structure. Infrared (IR) spectra were also calculated for the same systems. Raman and IR spectroscopy are complementary methods for analyzing the chemical composition of organic compounds. The Raman spectra obtained as a result of the calculation are shown in Figure 4.

Figure 4 shows that the Raman modes of these structures demonstrate noticeable peaks in two different frequency ranges of 1000–1600 cm^−1^ and 2100–2300 cm^−1^. The modes in different frequency ranges are explained by the different types of hybridization present in the system. In particular, sp-hybridized carbon atoms forming acetylene bonds mainly contribute to the relatively higher frequency range of 2100–2300 cm^−1^. At the same time, sp^2^-hybridized carbon atoms make a significant contribution in the relatively low-frequency ranges of 1000–1600 cm^−1^. In addition, the intensity of sp-induced combinational modes is usually greater than the intensity of sp^2^ hybridization. The oligomer O1 in the Raman spectrum has a peak at a frequency of ~3100 cm^−1^, which is responsible for the C-H present in the system. We have visualized the most perceptible vibrational modes. They can be found in the Supplementary Materials.

An additional analysis of the Raman spectra of the O1 oligomer and the 6,6,12-graphyne crystal indicates that for the O1 oligomer, the strong peak at a frequency of 2208 cm^−1^ corresponds to in-plane vibrations of triple inter-carbon covalent bonds that are adjacent to the central carbon bond l_6_ (see Figure 2 for covalent bond notation in oligomer O1). In this case, vibrations occur along the covalent bond without changing the valence angles. For the crystal structure, two main Raman peaks can be distinguished: at a frequency of 1235 cm^−1^ and at a frequency of 2159 cm^−1^. The first peak corresponds to the collective vibrations of carbon bonds l_6_ and l_1_, l_2_ (see Figure 3 for covalent bond notation in crystal). The triple bond l_3_ does not participate in such vibrations. The frequency 2159 cm^−1^, on the contrary, corresponds to the in-plane vibrations of triple bonds l_3_ and l_5_. Each bond oscillates along a selected straight line without changing the valence angles (see Supplementary Materials).

**Figure 5:**
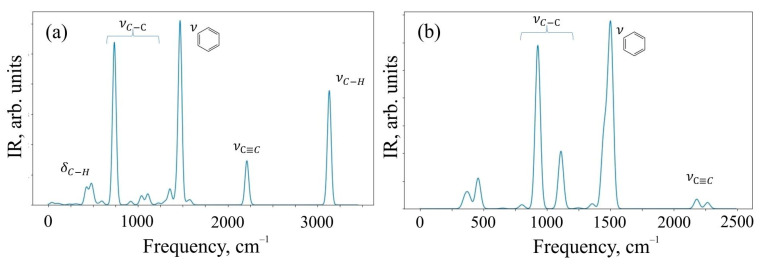
IR spectra of (**a**) O1 oligomer and (**b**) 6,6,12-graphyne crystal.

The results of the calculation of IR spectra are shown in Figure 5.

The spectral range of 400–900 cm^−1^ corresponds to deformation vibrations of simple C–H bonds in the O1 structure, and valence vibrations of C-H contribute to the frequency range of 3000–3200 cm^−1^, where a characteristic peak of intensity is observed. The range of 750–1200 cm^−1^ is characteristic of valence vibrations of simple C–C bonds of both structures. The peak observed at a frequency of 1500 cm^−1^ corresponds to the valence vibrations of aromatic (benzene) rings, and their deformation vibrations can be observed in the frequency range of 690–900 cm^−1^. The peak present in the spectra of both systems at a frequency of 2100 cm^−1^ is responsible for the valence oscillations of triple bonds C≡C present in the structures. We have also presented vibrational mode visualization for IR spectra in the Supplementary Materials.

Detailed analysis of the IR spectrum of the 6,6,12-graphyne crystal allows one to identify three characteristic peaks at frequencies of 929, 1111, and 1506 cm^−1^. The first two frequencies correspond to the so-called “zigzag” vibrations of single carbon bonds. In turn, the frequency 1506 cm^−1^ corresponds to the complex collective vibrations of the system, engaging bonds that form six-membered carbon rings composing the graphyne crystal (see Supplementary Materials). The IR spectrum of the O1 graphyne oligomer contains four clearly distinguishable strong peaks. The peak at a frequency of 737 cm^−1^ corresponds to low vibrations of C-C bonds outside the plane of the molecule, while carbon-hydrogen bonds are also involved. The second strong peak corresponds simultaneously to two close vibrational modes at frequencies of 1467 and 1473 cm^−1^. As in the case of the 6,6,12-graphyne crystal, these frequencies correspond to the covalent bond vibrations that form four benzene rings of the molecule. The third peak at a frequency of 2213 cm^−1^ corresponds to the vibrations of four triple inter-carbon bonds, which are designated as l_3_ in Figure 2. Finally, the highest frequency peak 3134 cm^−1^ as stated above corresponds to the vibrations of hydrogen atoms in the composition of benzene rings.

### 3.3. Thermal Stability

**Figure 6:**
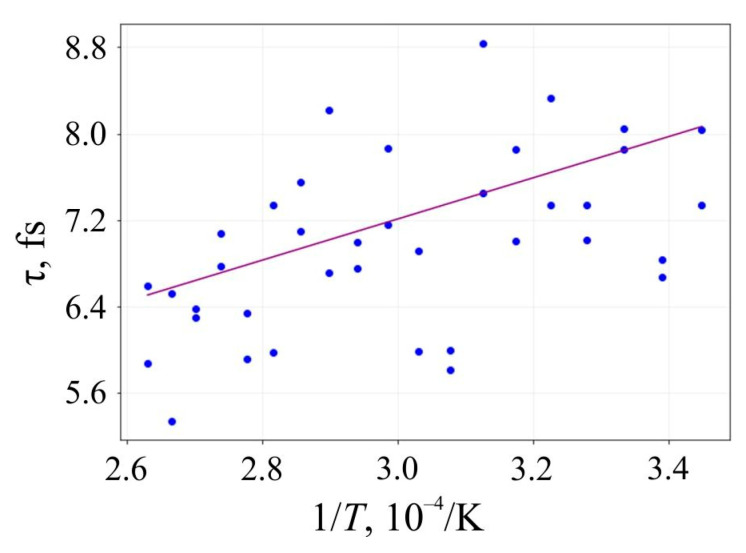
Lifetime of the 6,6,12-graphyne before its decomposition versus the inverse initial temperature T^−1^: circles are the results of the numerical calculation, and a solid line is the corresponding linear approximation obtained by the least-square method.

**Figure 7:**
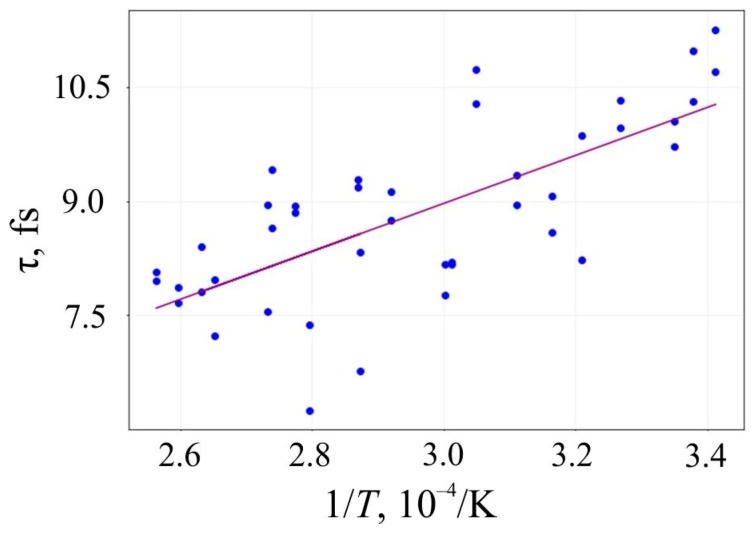
Lifetime of the oligomer O4 before its decay versus the inverse initial temperature T^−1^: circles are the results of the numerical calculation, and a solid line is the corresponding linear approximation obtained by the least-square method.

The lifetime *τ* of 6,6,12-graphyne and the smallest oligomer O4, which can be seen as the elementary building block of the entire crystal, up to their decomposition versus the inverse initial temperature T^−1^ for the initial temperatures T = 2500–4000 K, are presented in Figure 6 and Figure 7, respectively.

Approximating the inverse temperature dependence of *lnτ* with a straight line, we can determine the activation energy *E_a_* and the frequency factor *A* by the slope angle of this line and its intersection with the axis OY, respectively. These values and their standard deviations for 6,6,12-graphyne and the smallest 6,6,12-graphyne oligomer O4 are shown in Table 3. In addition, the estimates of the lifetime of the studied systems were predicted using the Arrhenius formula at two different temperatures: at room temperature (*T*_1_ = 300 K) and *T*_2_ = 800 K. These estimations are shown in Table 3.

The data were previously obtained for other two-dimensional carbon allotropes: for graphone, *E_a_* = 0.05 ± 0.01 eV and *A* = 10^13.5±0.1^ s^−1^ [34]; for graphane, *E_a_* = 1.74 ± 0.17 eV [35]; for graphene, *E_a_* = 4 eV [36].

The obtained results of the thermal stability study show that 6,6,12-graphyne (in the case of its synthesis) demonstrates high kinetic stability. The estimated lifetime at room temperature (300 K) gives a macroscopic “infinite” value, and at 800 K, the lifetime of structures is more than a microsecond. High thermal stability will allow the 6,6,12-graphyne crystals to be applied in a variety of applications without the use of extreme temperatures and refrigerants such as liquid nitrogen or helium.

## 4. Conclusions

The present work is devoted to an interesting two-dimensional carbon allotrope 6,6,12-graphyne, as well as some oligomers called radiaannulenes, which can be considered as the separate constituent fragments of such a crystal. In the framework of this study, we tried to find to what extent this material would be kinetically stable in the event of its successful synthesis. Molecular dynamics calculations using a nonorthogonal tight-binding model showed high thermal stability, both for fragment oligomers and for the entire crystal. Thus, according to our estimates, the stability of 6,6,12-graphyne concedes only to traditional graphene, exceeding the stability of such well-known materials as graphane and, even more so, graphone. In addition, we determined that Raman and infrared spectra were able to identify the 6,6,12-graphyne layer in a class of other two-dimensional carbon allotropes in the case of synthesis.

## Figures and Tables

**Table 1:** Bond lengths (Å) in the different oligomers and 6,6,12-graphyne. Bond lengths l_1_, l_2_, l_3_, l_4_, l_5_, l_6_, l_7_, and l_8_ are marked in Figure 2 and Figure 3. The values in parentheses represent the experimental bond lengths [19].

Structure	l_1_	l_2_	l_3_	l_4_	l_5_	l_6_	l_7_	l_8_
O1	1.461 (1.428)	1.451 (1.424)	1.247 (1.222)	1.452 (1.427)	1.246 (1.220)	1.437 (1.399)	-	1.094
O2	1.467 (1.434)	1.446 (1.418)	1.255 (1.226)	1.449 (1.423)	1.248 (1.233)	1.440 (1.402)	1.404 (1.360)	1.094
O3	1.467 (1.434)	1.446 (1.418)	1.255 (1.226)	1.449 (1.423)	1.248 (1.224)	1.441 (1.402)	1.404 (1.359)	1.094
O4	1.461	1.455	1.237	1.449	1.247	1.433	-	1.079
O5	1.462	1.455	1.237	1.448	1.247	1.433	-	1.079
O6	1.462	1.455	1.237	1.448	1.247	1.433	-	1.079
6,6,12-graphyne	1.456	1.425	1.254	1.456	1.245	1.428	-	-

**Table 2:** Values of the binding energy E_b_ (eV/atom) for the 6,6,12-graphyne crystal and its oligomeric structures O1, O2, O3, O4, O5, and O6.

Structure	Number of Carbon Atoms	E_b_ (eV/atom)
O1	38	5.64
O2	42	5.72
O3	64	5.73
O4	32	5.67
O5	54	5.71
O6	76	5.72
6,6,12-graphyne	∞	6.93

**Table 3:** Activation energy *E_a_* (eV), frequency factor *A* (s^−1^), and lifetimes (s) at *T*_1_ = 300 K and *T*_2_ = 800 K, determined from the Arrhenius equation for the crystal structures 6,6,12-graphyne and 6,6,12-graphyne-based oligomer O4.

Structure	*E_a_*, eV	*A*, s^−1^	*τ* (300 K), s	*τ* (800 K), s
O4	1.64 ± 0.2	10^14.3±0.5^	$\sim 10^{13}$	$\sim 10^{-4}$
6,6,12-graphyne	2.79 ± 0.2	10^15.3±0.5^	$\sim 10^{31}$	$\sim 10^{2}$

## Data Availability

The data presented in this study are available in the Supplementary Materials.

## Supplementary Materials

The following supporting information can be downloaded at: https://www.mdpi.com/article/10.3390/ma16051964/s1, Figure S1 contains information on valence angles in 6,6,12-graphyne-based oligomers [21]; Figure S2 contains information on valence angles between carbon bonds in 6,6,12-graphyne crystal.
