# Supplementary material for: Thermal Stability and Vibrational Properties of the 6,6,12-Graphyne-Based Isolated Molecules and Two-Dimensional Crystal
Source: Materials (Basel). 2023 Feb 27;16(5):1964. doi: 10.3390/ma16051964 (PMC10003780; doi:10.3390/ma16051964)
Supplement: Supplementary file 1 [file materials-16-01964-s001.zip › materials-2120988-supplementary.pdf]

materials

Supplementary Materials

for

Thermal Stability and Vibrational Properties of the 6,6,12-Graphyne-Based Isolated Molecules and Two-Dimensional Crystal

Ekaterina S. Dolina [1], Pavel A. Kulyamin [1], Anastasiya A. Grekova [1], Alexey I. Kochaev [2,3,4], Mikhail M. Maslov [1,2] and Konstantin P. Katin [1,2,*]

[1] Institute of Nanotechnologies in Electronics, Spintronics and Photonics, National Research Nuclear University "MEPhI", Kashirskoe Sh. 31, Moscow 115409, Russia
[2] Laboratory of Computational Design of Nanostructures, Nanodevices, and Nanotechnologies, Research Institute for the Development of Scientific and Educational Potential of Youth, Aviatorov Str. 14/55, Moscow 119620, Russia
[3] Laboratory of Acoustic Microscopy, Science Institute of Biochemical Physics Named after N.M. Emanuel of the Russian Academy of Sciences, Kosygina Str. 4, Moscow 119334, Russia
[4] Research and Education Center "Silicon and Carbon Nanotechnologies", Ulyanovsk State University, Leo Tolstoy Str. 42, Ulyanovsk 432017, Russia
* Correspondence: kpkatin@yandex.ru

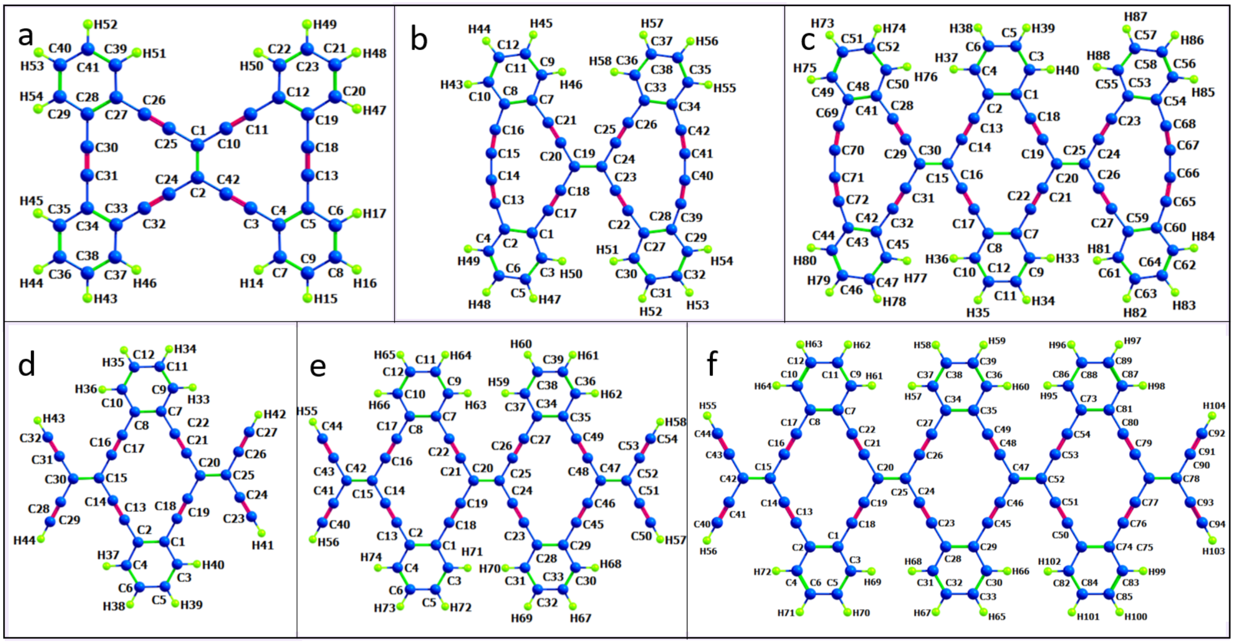

Figure S1. Valence angles of 6,6,12-graphyne-based oligomers: (a) O1: C(25)-C(1)-C(10) ***120.035***°; C(26)-C(25)-C(1) ***179.678***°; C(27)-C(26)-C(25) ***179.908***°; C(40)-C(41)-C(39) ***120.063***°; (b) O2: C(20)-C(19)-C(18) ***119.962***°; C(19)-C(20)-C(21) ***178.005***°; C(20)-C(21)-C(7) ***176.107***°; C(12)-C(11)-C(9) ***120.205***°; C(8)-C(16)-C(15) ***169.777***°; C(16)-C(15)-C(14) ***170.590***°;

C(15)-C(14)-C(13) ***170.568***°; C(14)-C(13)-C(2) ***169.699***°; (c) O3: C(29)-C(30)-C(31) ***119.918***°; C(30)-C(29)-C(28) ***178.000***°; C(41)-C(28)-C(29) ***176.197***°; C(16)-C(15)-C(14) ***120.153***°; C(15)-C(14)-C(13) ***179.769***°; C(14)-C(13)-C(2) ***179.941***°; C(6)-C(5)-C(3) ***120.043***°; C(48)-C(69)-C(70) ***169.744***°; C(69)-C(70)-C(71) ***170.508***°; C(70)-C(71)-C(72) ***170.513***°; C(71)-C(72)-C(43) ***169.762***°; (d) O4: C(26)-C(25)-C(24) ***120.058***° [*116.02*°]; C(21)-C(20)-C(19) ***120.183***° [*117.45*°]; C(20)-C(21)-C(22) ***179.808***° [*174.6*°]; C(21)-C(22)-C(7) ***179.892***° [*177.8*°]; C(20)-C(19)-C(18) ***179.927***° [*178.6*°]; C(19)-C(18)-C(1) ***179.769***° [*176.56*°]; C(12)-C(11)-C(9) ***120.069***°; (e) O5: C(53)-C(52)-C(51) ***120.050***° [*115.74*°]; C(25)-C(26)-C(27) ***179.569***° [*179.30*°]; C(26)-C(27)-C(34) ***179.878***° [*175.8*°]; C(25)-C(24)-C(23) ***179.530***° [*176.52*°]; C(24)-C(23)-C(28) ***179.917***° [*177.9*°]; C(26)-C(25)-C(24) ***119.969***° [*117.91*°]; C(47)-C(48)-C(49) ***179.683***° [*171.3*°]; C(48)-C(49)-C(35) ***179.782***° [*177.5*°]; C(47)-C(46)-C(45) ***179.723***° [*178.8*°]; C(46)-C(45)-C(29) ***179.743***° [*174.2*°]; C(48)-C(47)-C(46) ***119.737***° [*117.59*°] C(12)-C(11)-C(9) ***120.054***°; (f) O6: C(91)-C(90)-C(93) ***120.032***°; C(90)-C(91)-C(92) ***179.879***°; C(77)-C(78)-C(79) ***119.738***°; C(78)-C(79)-C(80) ***179.720***°; C(79)-C(80)-C(81) ***179.778***°; C(88)-C(89)-C(87) ***119.980***°. The values in square brackets correspond to the experimentally obtained data (see Ref. 22).

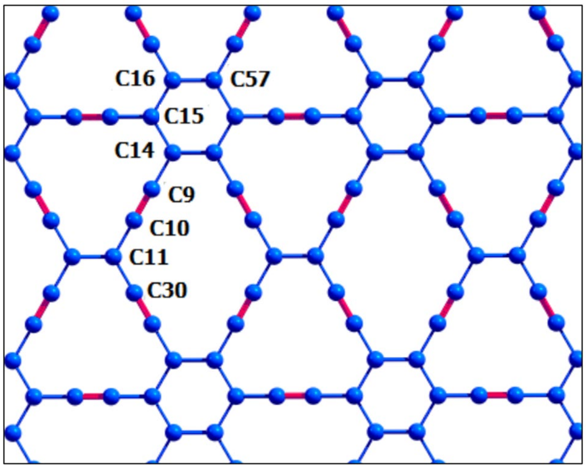

Figure S2. Valence angles of 6,6,12-graphyne crystal: C(30)-C(11)-C(10) ***120.384***°; C(11)-C(10)-C(9) ***179.952***°; C(10)-C(9)-C(14) ***179.578***°; C(15)-C(16)-C(57) ***120.315***°.
